# Rural–urban migration and mental and sexual health: a case study in Southwestern China

**DOI:** 10.1080/21642850.2013.839384

**Published:** 2013-12-20

**Authors:** Xiushi Yang

**Affiliations:** ^a^ Department of Sociology and Criminal Justice, Old Dominion University, Norfolk, VA, USA

**Keywords:** mental health and disorder, sexual and reproductive health, social determinates of health, observational research, immigrants or migrants

## Abstract

Massive rural–urban temporary migration has taken place amid China's rapid economic growth and development. Much has been written about the economic causes and consequences of this massive migration; less studied are the potential health and behavioral impacts of migration on migrants. Using data from a population-based sample survey conducted in southwestern China, this paper examines the potential impact of rural–urban migration and post-migration urban living on migrants' mental health and sexual risk behavior. The results suggest that regardless of places of origin and destination temporary migrants had on average poorer mental health and riskier sexual behavior than non-migrants. Compared to living in rural areas, living in urban areas does not make statistical difference in residents' mental health; it is only marginally associated with riskier sexual behavior. Rural–urban temporary migrants' mental health and health risk sexual behavior deserve more immediate research attention. Both selectivity of temporary migrants and migration-induced psycho-socio-behavioral changes may have contributed to migrants' poorer mental health and riskier sexual behavior. However, more theory-driven research with longitudinal design is needed before firm conclusions can be drawn about the underlying mechanisms that mediate or moderate the impact of temporary migration on migrants' mental health and sexual risk behavior.

## Introduction

Massive rural–urban migration that involves no change in migrants' official residence registration in China, typically referred to in the literature as temporary migration, has been a major demographic response to the rapid economic growth and development since the 1980s. Although varied by sources and classifications, temporary migrants, who live and work in a place other than their officially registered residence, have grown from 11 million in 1982 to over 260 million in 2010 (Liang & Ma, 2004; National Bureau of Statistics of China, 2012). The number is estimated to further grow to over 300 million by 2025. This is almost the population size for the entire USA. The uprooting and on the move of so many people will no doubt have far reaching impact on the economic and social lives of Chinese society.

Much has been written about the economic causes and consequences of this massive migration in contemporary China. Rural–urban migrant labor has played a vital role in meeting the expanding yet fluctuating urban labor demand and contributing to China's phenomenal economic growth since the 1980s. While research also suggests that temporary migrants often are economically marginalized and socially isolated and experience social and structural discriminations in the places of urban destination, which renders them vulnerable to health and health-related behavioral problems (Li, Stanton, Fang, & Lin, 2006; Lin et al., 2011; Sudhinaraset, Mmari, Go, & Blum, 2012; Yang, Derlega, & Luo, 2007; Yang & Xia, 2008), the impact of temporary migration on migrants' health in China is less studied. Limited research on temporary rural–urban migrants' mental health in China has actually produced mixed evidence (Li et al., 2007; Lin et al., 2011; Wong, He, Leung, Lau, & Chang, 2008). Further, prior research on migrant health and behavior does not always use the appropriate comparison group (Lu, 2010) or distinguish the impact of being a migrant from that of living in an urban place (Yang & Luo, 2009). Rural–urban migrants are subject to the simultaneous influence of the process of migration and post-migration urban living; it is important to separate the impact of migration from that of post-migration urban living. Theoretically, it helps to better understand why and what actually contributes to migrants' poorer health and riskier sexual behavior. Pragmatically, it helps to better guide the development of policy and/or intervention programs to alleviate migrants' health and behavioral problems. Failure to disentangle the two sources (migration vs. urban living) of health and behavioral influences can limit our understanding of the migration and health/behavior links and in turn hamper our ability to respond to potential health challenges faced by rural–urban temporary migrants.

Using data from a population-based probability sample survey in China's Yunnan province, this paper examines the impact of rural–urban temporary migration on migrants’ mental and sexual health. The goal is to answer two critical questions: (1) are rural–urban temporary migrants more likely to experience mental health problems and sexual risk behavior than comparable non-migrants and (2) if they are what contributes to migrants’ poorer mental health and riskier sexual behavior: the process of migration (i.e. being migrant), urban environment (i.e. post-migration urban living), or both. Findings will contribute to the migration and health literature in China by providing empirical evidence on the extent of rural–urban temporary migrants' mental/sexual health problems and the relative importance of impact of migration vs. post-migration urban living on migrants' health problems.

## Migration and mental/sexual health

There is extensive literature on the link between international migration and mental health (Bhugra, 2004; Rechel, Mladovsky, Ingleby, Mackenbach, & McKee, 2013). Although less studied, internal rural–urban migration in developing countries arguably is just as stressful and affects migrants' mental health in similar ways as international migration (Lu, 2010). Despite the initial physical health advantage found among rural–urban migrants (Chen, 2011; Hesketh, Ye, Li, & Wang, 2008; Lu, 2008), rural–urban migrants are subject to multiple sources of stress, which compromises their mental health (Cui, Rockett, Yang, & Cao, 2012; He & Wong, 2013; Lu, 2010; Wong et al., 2008; Yang et al., 2012) and renders them vulnerable to sexual risk behaviors (Wang, Wei, et al., 2010; Yang, 2006; Yang et al., 2007; Yang & Xia, 2008; Zhang, Chow, Jahn, Kraemer, & Wilson, 2013). First, temporary migration detaches migrants from their supporting family/social networks and results in disruption of family and sexual life as well as loss of familiar living/social environments. The separation from family and social networks is particularly challenging, becoming “an unremitting source of anxiety” for most temporary rural–urban migrants (Jochelson, Mothibeli, & Leger, 1991). It also creates sort of behavioral control vacuum, whereby temporary migrants feel free from family monitoring and less constrained by social norms since what they do while away from home can remain largely anonymous (Yang, 2006). Both likely contribute to migrants’ greater likelihood of risky sexual behavior.

Second, rural–urban temporary migrants are concentrated in the margins of the urban economy and with few exceptions do the dirty, dangerous, and dead-end jobs, shunned by urban natives (Knight, Song, & Jia, 1999; Solinger, 1999; Wang, Zuo, & Ruan, 2002). While the economic reforms since the 1980s have provided market alternatives to government provision of employment and services in urban China and whereby made it possible for migrants to live in cities without having a local residence registration or *hukou* (Chan, 1996; Yang, 1993), the lack of local urban *hukou* remains a formidable structural barrier for rural–urban temporary migrants to access mainstream economic opportunities and social services (Chan, Liu, & Yang, 1999; Wang, 2004). This man-made divide between temporary migrants and urban natives effectively perpetuates the economic marginalization of temporary migrants in Chinese cities, which in turn contributes to migrants’ poor mental health and sexual risk behavior (Wong, Chang, & He, 2007).

Third, with few exceptions most rural–urban temporary migrants in China are socially, culturally, and residentially isolated from the “mainstream” society. Because of their lack of local urban *hukou* and poor rural background, rural–urban temporary migrants are often looked down at and experience both institutional discrimination and social stigma (Lin et al., 2011; Wang, Li, Stanton, & Fang, 2010; Wen & Wang, 2009). Further, most migrants live with fellow villagers/migrants at the place of work or in rural–urban transitional neighborhoods characterized by overcrowding, social disintegration, and lack of social and health services (Ma & Xiang, 1998; Zhang, 2001). Migrants' social interaction in the city often does not go beyond that with fellow villagers or migrants. Despite limited economic gains as compared to conditions in their rural origins, most rural–urban temporary migrants experience little social or cultural assimilation in the city and feel helpless, insecure, discontented, and resentful, all of which render them vulnerable to mental health problems and risky sexual behaviors (Anderson, Qingsi, Hua, & Jianfeng, 2003).

## Urban living and mental/sexual health

Despite its many advantages, urban living has been associated with poorer mental health (Paykel, Abbott, Jenkins, Brugha, & Meltzer, 2000; Peen, Schoevers, Beekman, & Dekker, 2010) and health risk sexual behavior (Berhan & Berhan, 2013). Conceptual work that links urban living to health and health-related behaviors has generally focused on the physical and socioeconomic characteristics of the urban environment (Galea & Vlahov, 2005; Galea, Uddin, & Koenen, 2011; Moore, Gould, & Keary, 2003).

The physical characteristics of the urban environment expose residents to multiple stressors and other health compromising conditions, including crowding/high density, crimes/personal safety concerns, high-rise housing, increased noise level, air pollution, traffic jams, poor housing and built environment, and poor or inaccessible health and social services (Evans, 2003; Macintyre, Maciver, & Sooman, 1993). Although improved public health measures and medical innovations have largely separated population concentration in cities from infectious diseases that killed millions of city dwellers in historical times and during the early industrialization in Europe and North America (Rosen, 1993), they have not done much in improving the physical health hazards. On the contrary, partly due to poor planning, recent rapid urbanization in developing countries, including China, has not only replicated many of the physical hazards associated with urban living but even created similar poor sanitation and squalid living conditions in transitional neighborhoods or peri-urban slums in many cities (Weiss & McMichael, 2004). These physical characteristics of cities, along with heightened competition in cities, make urban living more stressful, affecting both directly and indirectly the mental health of urban residents (Evans, 2003; Galea, Freudenberg, & Vlahov, 2005; Marsella, 1998).

The socioeconomic characteristics of the urban environment can be characterized by more pronounced inequalities by social groups and neighborhoods, greater interpersonal tension and violence, and weakened social cohesion and personal control, all contributing to increases in mental health problems (Evans, 2003; Mair, Diez, & Galea, 2008). While these stress-producing socioeconomic characteristics of the urban environment could affect all urban residents, the poor and socioeconomically underprivileged may bear disproportionately the mental health burden. Living under poverty and in distressed neighborhoods, along with perceived inequalities/injustice and feelings of helpless about them, could be particularly detrimental to mental health among the socioeconomically disadvantaged urban residents, including rural–urban migrants (Evans, 2003). Research in China has also suggested that rural–urban temporary migrants experience additional psychological stress associated with adaptation and acculturation (Chen, 2011; Yang et al., 2012) as well as perceived and experienced social stigma and discrimination (Lin et al., 2011; Wang, Li, et al., 2010; Wen & Wang, 2009).

Both physical and socioeconomic characteristics of the urban environment also can contribute to health risk sexual behavior (Cohen et al., 2000; Frye et al., 2006). The physical characteristics of the urban environment important to understanding sexual risk behaviors include the exposure and access to extramarital/commercial sex and the quality of the built environment. The existence of more commercial sex outlets in urban areas affects directly the access to and costs of sexual risk behavior (Yang & Luo, 2009). The quality of the neighborhood environment can determine the extent to which its residents are economically marginalized and socially isolated, which in turn influences behavior and contributes to sexual risk behavior (Cohen et al., 2000).

Socioeconomic characteristics of the urban environment may also be conducive to the spread of risky sexual behaviors (Frye et al., 2006). Like any other human behavior, risky sexual behaviors are not inborn but learned through socialization (Bandura, 1986; Clark, 1987). Individuals learn to behave socially by interpreting images or messages they receive in social interactions or in public domains about what is socially acceptable and by observing and imitating the behavior of others they come into contact. In particular, social norms about sex and sexual networks play an important role in influencing sexual risk behavior (Frye et al., 2006; Galea, Ahern, & Vlahov, 2003; Latkin, Forman, Knowlton, & Sherman, 2003; Richard, Bell, & Montoya, 2000).

Urban living also is associated with greater anonymity and residential mobility, erosion of traditional values, which leads to more liberal behavioral norms, and increased diversities in population and social networks (Frye et al., 2006; Galea et al., 2005; Weiss & McMichael, 2004). These features, along with greater exposure to sex-related cultural, social, and physical scenes in cities, may lead to more tolerable attitude toward sexual risk behavior. The more tolerable normative environment, reinforced by the presence of people who practice sexual risk behaviors, can facilitate the spread of sexual risk behavior in cities (Yang, 2005). The breakdown in traditional norms about sexual behavior in China in the last three decades is arguably one of the main contributing factors of the spread of commercial sex and other risky sexual behaviors in urban China (Gil, Wang, Anderson, Lin, & Wu, 1996; Hyde, 2000; Zheng et al., 2011).

## Research setting

Data used in the analysis are from a population-based survey conducted in 2003 in China's Yunnan province. The province is located in southwestern China, bordering Myanmar and home to the largest number of ethnic minority populations in the country. Compared to the east coastal region, Yunnan remains to be less developed with limited direct foreign investments. In 2012, 36.8% of its population was classified as urban residents, making Yunnan one of the three least urbanized provinces in China (National Bureau of Statistics of China, 2012).

Like the rest of China, Yunnan province has experienced a large increase in rural–urban temporary migration. In fact, with a growth rate of 230% between 1990 and 2000, it was the only province away from the east coast that had more than doubled its temporary migrant population during the period (Liang & Ma, 2004). Besides structural changes, tourism has been the other driving force in the rapid growth of temporary migrant population in the province, which has become one of the most attractive destinations for both domestic and international tourists. Tourism, interacting with market and social changes, may also have fueled the growth and spread of commercial sex in Yunnan (Hyde, 2000).

## Sampling and data collection

Sample selection of the survey followed a three-stage sampling procedure. Details of the sampling design were described elsewhere (Yang & Xia, 2008). Briefly, all counties/cities in Yunnan were ranked according to a combination of aggregate statistics on numbers of HIV/AIDS cases, registered drug users, and temporary migrants; from the ranked lists, four counties and four cities were selected. The selection was not random but gave priority of selection to counties/cities ranked high on the lists and geographically representing the province. Next, in each of the eight selected counties/cities, all townships in rural areas and all neighborhoods in cities were ranked according to their aggregate statistics on HIV cases, drug users, and temporary migrants. From the ranked lists, five townships and/or neighborhoods were selected from each selected county/city. Again the selection was not random but gave priority of selection to townships/neighborhoods ranked high on the lists and geographically representing the selected county/city. The first two stages of the sampling resulted in a total of 40 townships and neighborhoods (8 selected counties/cities × 5 townships/neighborhoods from each).

In the third and final stage, all individuals 18–55 years of age were listed by townships/neighborhoods in one of four mutually exclusive categories: HIV positive, drug users, temporary migrants, and non-migrants. If an individual appeared in more than one category, the individual was reassigned to only one category according to the following priority order: HIV, drug user, migrant, and non-migrant. From each of the four mutually exclusive listings, disproportionate probability sampling (Bilsborrow, Hugo, Oberai, & Zlotnik, 1997) then was used to select a target systematic random sample of up to 20 HIV positive, 30 drug users, 40 temporary migrants, and 60 non-migrants, respectively. Probability of selection was calculated as the ratio between the actual number of individuals selected from each list and the total that was available on the list. Sampling weights, which were calculated as the inverse of the probability of selection, were applied to arrive at a final analytic sample representative of the populations in the 40 selected townships and/or neighborhoods.

During the fieldwork, trained interviewers visited the sampled individuals, explained to them the purpose of the study, their right to refuse, and compensation for their time, and invited them to participate. If the respondent was absent, a second visit was scheduled. If a respondent could not be reached the second time or refused to participate, a replacement was selected randomly from the original sampling list containing the absent or refused respondent. Of the original sample of 5570 individuals, 5382 consented to participate and completed a face-to-face interview, which took place in private at the respondent's home or if he/she preferred, a place away from home. All interviews were conducted in Mandarin or the respondent's dialect if the respondent could not communicate in Mandarin.

## Statistical methods

Version 12 of the STATA software is used to conduct statistical analyses. Data analysis focuses on the impact of being temporary migrant and/or urban residence on respondents’ mental health and sexual risk behavior and is divided into two components. The bivariate analysis compares measures of mental health and sexual risk behavior between temporary migrants and non-migrants and between urban and rural residents. Independent sample *t* tests of difference in means are conducted to see if the between-group differences in mental health and sexual risk behavior are statistically significant.

Multiple linear regressions are then used to examine the independent impact of being temporary migrant and urban living on mental health and sexual risk behavior while controlling for socio-demographic characteristics and other potential confounding variables. The goal is to gain further insight into the relative importance of the process of migration vs. urban living on rural–urban temporary migrants’ mental health and sexual risk behavior. The multiple regression analysis also helps to shed light on whether the process of migration and urban living affects temporary migrants’ mental health and sexual risk behavior independently or interactively as well as on other important correlates of mental health and sexual risk behavior. All analyses use the “svy” methods in STATA, which take into consideration of sampling probability and survey design as well as the clustering effect of the sample (i.e. the intra-correlation among respondents from the same city/township/neighborhood).

## Measures

The dependent variables are measures of mental health and sexual risk behavior. Mental health was measured by the Center for Epidemiologic Studies Depression Scale (Radloff, 1977). It was based on ratings of 20 statements on a four-point scale on the frequency of depressive symptoms the respondent experienced in the week prior to the interview. Answers to the 20 statements were summed to form the “depression” scale (Cronbach's *α* = 0.84). The higher the scale, the more depressed the respondent felt. Sexual risk behavior was measured by a composite risky Sexual Behavior Index. The index was constructed by summing non-missing responses (1 for yes and 0 for no) across eight dichotomous sexual behaviors in the 30 days prior to the interview (e.g. had unprotected casual sex; involved in commercial sex; had more than one casual sex partner; and was drinking while having sex) with equal weight. Research suggests that an index combining multiple behaviors may be a more accurate measure than any single dichotomous measure (Williams et al., 2001). The higher the resulting index value, which ranges from 0 to 8, the more risky the respondent's sexual behavior (Cronbach's *α* = 0.80).

The main independent variables are temporary migrant status and urban residence. Temporary migrant is defined as someone who did not possess the official local household registration in the township/city where they lived and were interviewed. To further ascertain potential differences by migrants’ places of origin, temporary migrants were further classified by rural–urban origins; temporary migrant status was thus represented by two dummy variables, indicating temporary migrants with rural and urban origins, respectively, in the analyses. Urban residence was defined as residence in all cities and officially established urban towns.

The control variables, which may confound or mediate the association between migrant status and/or residence and mental health or sexual risk behavior, include individual socio-demographic characteristics and index/scale measures of economic marginalization, social influence of sexual risk behavior, and felt social control over behavior, all expected to be associated with being temporary migrant and/or urban living. All individual demographic characteristics are self-explanatory. The Economic Marginalization Index was constructed by first dichotomizing answers (1 vs. 0) to 15 questions on employment (unemployed vs. employed), industry (agriculture, construction, and personal services vs. others), ownership of company where employed (self/small privately owned vs. state/collective owned), occupation (menial jobs, including farmers and personal service workers, vs. more prestigious ones), monthly income (under 600 yuan vs. 600 yuan or higher), perceived income level and working conditions (below average vs. average or better), and eight employment-related benefits (not having vs. having pension, health insurance, social security, unemployment benefit, paid holidays, paid sick leave, housing allowances, and on-job training), and then summing the 0/1 answers. The resulting index ranges in value from 0 to 15; the higher the index the more economically marginalized the respondent (Cronbach's *α* = 0.86).

For social influence, respondents reported separately on whether they knew if parents, siblings, relatives, and friends had multiple sexual partners, same sex behavior, and exchanged sex for money. The 12 member-behavior pair-wise answers were then summed to form a Sexual Behavior Influence Index. The index ranges between 0 and 12 in values; the higher the index value the greater the social influence of sexual risk behavior. For felt social control over behavior, two indirect measures were used. The first was a lax Social Control Index, which was based on a modified version of the Deviance and Attitudes toward Authority Scale (Emler, 2005). Respondents reported yes (1) or no (0) on their personal experience with nine events indicating disrespect for laws or use of “deviant” ways to achieve personal ends (e.g. I have deliberately traveled on a train/bus without a ticket; I have stolen bicycle(s) from streets). Answers were then summed to create the lax Social Control Index, which ranges between 0 and 9. The higher the index value, the more likely the respondent had behaved in disrespect for laws or deviant ways, indicating indirectly lax social control (Cronbach's *α* = 0.71). The second indirect measure used was a dichotomous variable on respondents’ living arrangement, which took the value of 1 if the respondent lived alone and 0 if he/she lived with family or other relatives or friends. Living alone was assumed to be associated with more lax social control over one's behavior.

## Results

At the bivariate level, data in Table 1 show that regardless of places of origin temporary migrants scored significantly (*p* < .05) higher than non-migrants on the depression scale. The differences in sexual risk behavior between temporary migrants and non-migrants were even more significant (*p* < .01) and more pronounced. On average, the Sexual Risk Behavior Index values for temporary migrants (0.54 and 0.63, respectively) were at least three times that of non-migrants (0.18). However, differences in average depression scale or Sexual Risk Behavior Index scores between urban and rural residents were small and statistically not significant.

**Table 1.** Mental health and sexual risk behavior by migrant status and by residence.^a^

	Depression scale (range 20–80)	Sexual Risk Behavior Index (range 0–8)
*Migrant status*		
Non-migrant	32.97	0.18
Temporary migrant with rural origin	34.91*	0.54**
Temporary migrant with urban origin	34.85*	0.63**
*Residence*		
Urban	33.06	0.24
Rural	33.43	0.17
Total sample	33.14	0.22

^a^Results are based on “svy” methods in STATA and adjusted for sampling probability and survey design, including intra-correlation (or clustering effect) among respondents from the same township/neighborhood. Statistical significance is based on comparison across the three migrant and non-migrant groups and between urban and rural residence, respectively.

**p < .01; *p < .05.

In terms of average individual demographic, economic, and psychosocial characteristics, which may be correlated with mental health and/or sexual risk behavior and consequently could confound the bivariate comparisons presented in Table 1, data in Table 2 again showed more significant and pronounced differences by temporary migrant status than that characterize rural and urban comparisons. Regardless of places of origin, temporary migrants were on average younger but less educated and less likely to be married than non-migrants. Economically they were more marginalized than non-migrants. Temporary migrants also scored significantly higher on the loneliness scale, lax Social Control Index, and Sexual Influence Index, and were significantly more likely to live alone than non-migrants. By contrast, in only two out of the nine characteristics examined, did urban residents differ significantly from their rural counterparts. On average, urban residents were better educated but experienced less economic marginalization than rural residents.

**Table 2.** Individual demographic, economic, and psycho-socio-behavioral characteristics by migrant status and by residence.^a^

		Migrant status			Residence	
	Total sample	Migrant rural	Migrant urban	Non-migrant	Urban	Rural
*Demographic characteristics*						
Male (%)	51.4	50.5	55.7	51.0	50.6	54.2
Age	32.6	29.5	28.1	33.0**	32.7	32.3
Education^b^	2.9	2.4	2.9	3.0**	3.1	2.4**
Currently married (%)	81.6	60.2	57.7	83.9**	80.8	84.4
*Economic characteristics*						
Marginalization Index	9.8	10.7	10.5	9.8*	9.5	11.1**
*Psycho-socio-behavioral characteristics*						
Loneliness scale	37.2	40.7	39.9	36.9**	37.0	37.8
Lax Social Control Index	0.4	0.5	0.6	0.4**	0.4	0.5
Living alone (%)	3.5	18.5	20.7	1.8**	3.7	2.6
Sexual Influence Index	0.2	0.4	0.5	0.2**	0.2	0.2

^a^See note a in Table 1.

^b^Educational attainment is an ordinal variable: 1 illiterate or semi-illiterate; 2 elementary school; 3 junior high school; 4 senior high school; 5 vocational school; 6 two/three years college; and 7 four years college or more.

**p < .01; *p < .10.

Given the significant differences, particularly by migrant status, in demographic, economic, and psychosocial characteristics and the potential association between these individual characteristics and mental health, sexual risk behavior, or both, the analyses now move to multiple regressions that try to control for their potential confounding effects on the observed association between migration/urban residence and mental health and sexual risk behavior.

Table 3 presents multiple regression analyses on mental health as measured by the depression scale in three different model specifications. In Model 1, the depression scale was regressed on migrant status and type of residence only. Consistent with the bivariate results in Table 1, the results suggested that regardless of places of origin and destination, temporary migrants scored significantly higher on the depression scale than non-migrants. However, living in an urban area (as compared to living in a rural area) made no significant difference in self-reported depression symptoms. The explanatory power of the model overall was weak; the two primary independent variables explained only about 1% of the variances in the depression scale.

**Table 3.** Regression analysis of depression symptoms in the week prior to the interview.^a^

	Dependent variable: depression scale		
Independent/control variables^b^	Model 1	Model 2	Model 3
*Independent variable*			
Temporary migrant with rural origins	1.98***	1.03*	−0.43
Temporary migrant with urban origins	1.93**	1.30*	−0.27
Urban residence	−0.41	−0.03	0.23
*Demographic characteristics*			
Male	–	−1.34***	−1.32***
Age	–	<−0.01	<−0.01
Education^c^	–	−0.63***	0.08
Currently married	–	−2.49***	−0.79***
*Economic characteristics*			
Marginalization Index	–	–	0.05
*Psycho-socio-behavioral characteristics*			
Loneliness Scale	–	–	0.55***
Lax social control scale	–	–	0.93***
Living alone	–	–	0.16
Unweighted sample size	5442	5393	5342
Model *R*^2^	0.01**	0.05***	0.42***

^a^See note a in Table 1.

^b^The reference categories for the dummy variables of temporary migrants, urban residence, male, currently married, and living alone are non-migrant, female, single, and living with others, respectively.

^c^See note b in Table 2.

*p < .10; **p < .05; ***p < .01.

When the demographic characteristics were controlled for in Model 2, the coefficients for the two migrant variables were considerably reduced in both size and significance level. In fact, both coefficients were only marginally significant at the 10% level. Among the demographic characteristics, gender, education, and marital status were all significant correlates of depression symptoms. Being male, less educated, and married were all negatively associated with the depression scale. Overall, Model 2 explained more variances (5%) in the depression scale than Model 1.

In Model 3, in addition to demographic characteristics, economic and psychosocial characteristics were also controlled for. With the additional controls, the coefficients for the migrant status variables were not only drastically reduced in size, but also reversed in sign and lost their statistical significance as correlates of the depression scale. Among the economic and psychosocial characteristics, Economic Marginalization Index did not obtain statistical significance. Lonely feelings (measured by the loneliness scale) and experiences of lax social control (measured by the lax Social Control Index) were both highly significant in their association with depression. In terms of explanatory power, this full model was far superior over the two reduced models, explaining 42% of the variances in the depression scale.

Table 4 presents multiple regression analyses on sexual risk behavior by the same three different model specifications. When the Sexual Risk Behavior Index variable was regressed on migrant status and type of residence only in Model 1, regardless of places of origin and destination, temporary migrants were significantly riskier sexually than their non-migrant counterparts. However, urban living made no difference statistically in individuals' sexual risk behavior.

**Table 4.** Regression analysis of sexual risk behavior in the month prior to the interview.^a^

	Dependent variable: Sexual Risk Behavior Index		
Independent/control variables^b^	Model 1	Model 2	Model 3
*Independent variable*			
Temporary migrant with rural origins	0.35***	0.30***	0.22***
Temporary migrant with urban origins	0.44***	0.39***	0.25***
Urban residence	0.06	0.07	0.08*
*Demographic characteristics*			
Male	–	0.13***	0.08**
Age	–	<−0.01	<−0.01
Education^c^	–	−0.03*	−0.01
Currently married	–	−0.17**	−0.03
*Economic characteristics*			
Marginalization Index	–	–	<0.01
*Psycho-socio-behavioral characteristics*			
Loneliness Scale	–	–	0.01**
Lax social control scale	–	–	0.16***
Living alone	–	–	0.29***
Sexual influence	–	–	0.18***
Unweighted sample size	5442	5393	5342
Model *R*^2^	0.03***	0.04***	0.11***

^a^See note a in Table 1.

^b^The reference categories for the dummy variables of temporary migrants, urban residence, male, currently married, and living alone are non-migrant, female, single, and living with others, respectively.

^c^See note b in Table 2.

*p < .10; **p < .05; ***p < .01.

When the four demographic characteristics were controlled for in Model 2, the coefficient estimates for the two migrant variables hardly changed in size and remained highly significant. Regardless of places of origin and destination, being temporary migrant was positively associated with significantly riskier sexual behavior. The coefficient estimate for urban residence also was little changed; urban living remained an insignificant factor in understanding individual sexual behavior. Of the four demographic characteristics, age did not make much difference in people's sexual risk behavior, while gender, education, and marital status all were significant correlates of individuals' sexual risk behavior. Being male was associated with riskier sexual behavior, but having more education and being married appeared to afford significant protection against sexual risk behavior.

With the additional controls for differences in economic and psycho-socio-behavioral characteristics in Model 3, the coefficients for the two migrant variables were reduced in size but remained highly significant. Further, the coefficient for the urban residence also gained statistical significance (*p* = .087). With all control variables in the model, living in an urban area was associated with riskier sexual behavior than living in rural areas. Of the demographic characteristics, only gender remained a significant correlate of sexual risk behavior. All four psycho-socio-behavioral characteristics were statistically significant in influencing sexual risk behavior. Other things being equal (controlled for), lonely feelings, experiences of lax social control, and living alone were all important risk factors for sexual risk behavior. Perceived sexual risk behaviors among respondents' social network members also exerted significant and positive impact on respondents' own sexual risk behavior. Overall, the full model explained 11% of the sample variances in sexual risk behavior, more than two times more than that explained by the two reduced models (i.e. Models 1 and 2).

## Discussion and conclusions

As more and more rural Chinese migrate to cities amid the country's rapid development and urbanization, the health consequences of rural–urban temporary migrants have attracted much attention and concerns from both scholars and policy-makers. There is growing consensus that migrants are more vulnerable to mental health problems and sexual risk behavior. Less understood is whether migrants' increased vulnerability results from the process of migration (being migrant), post-migration urban living, or both. Literature on rural–urban migration and migrant health has generally not been specific about the separate impact of post-migration urban living, while literature on urban health in general, which is still limited in China, has not been particularly concerned with migrants or the potential behavioral impact of urban living. In this paper, we explore the impact of temporary migration and post-migration urban living on migrants' mental health and sexual risk behavior with the focus on the potentially different and/or joint impact of being migrant and urban living.

The results suggested that at the bivariate level, temporary migrants, regardless of their places of origin and destination, had on average poorer mental health and health riskier sexual behavior than non-migrants. Compared to rural residence, living in urban areas, however, did not make much of a difference in respondents' mental health or sexual risk behavior. As a group, temporary migrants also differed significantly from non-migrants in eight out of nine demographic, economic, and psycho-socio-behavioral characteristics that also were related to mental health and/or sexual risk behavior. By contrast, urban residents differed from rural residents only in the average level of education received and in the index measure of economic marginalization.

When the other individual characteristics were controlled for in the multiple regressions, the results revealed that temporary migrants were no longer significantly different from non-migrants in self-reported depression symptoms. Meanwhile, being male and married were two independent and significant protective factors in respondents’ mental health, while social isolation (measured by the lonely feeling scale) and experiences of lax social control were two independent and significant risk factors of respondents’ mental health. Together, the results suggest that for mental health it may not be so much of the process of migration but more importantly the selectivity of temporary migrants in demographic and psycho-socio-behavioral characteristics that may predispose them to mental health problems. This does not completely rule out any potential detrimental impact of migration on migrants’ mental health. On the contrary, economic marginalization, lonely feelings or social isolation, and experiences of lax social control all are as much of the result of migrant selectivity as the result of post-migration mal-adaptation of migrants. As such, the results also suggest that the potential detrimental impact of migration on migrants’ mental health may be mediated through their migration-induced psycho-socio-behavioral changes. Future research is needed, which employs more rigorous study design to collect pre- and post-migration or longitudinal data that allow tests of changes in psycho-socio-behavioral characteristics over time as well as any roles they play in mediating the relationship between migration and migrants’ mental health.

For sexual risk behavior, the control of the other individual characteristics only reduced the extent of migration's impact. Temporary migrants, regardless of their places of origin and destination, remained significantly riskier than non-migrants in sexual behavior. Of the four demographic characteristics, only gender remained statistically significant. Compared to comparable females, males were significantly associated with riskier sexual behavior. This somewhat contradicted earlier findings with the same data-set when males were found no riskier than females (Yang & Luo, 2009) or even less risky sexually than females (Yang & Xia, 2006, 2008). However, the earlier studies used a single indicator (e.g. having casual or commercial sex in the prior 30 days) to measure sexual risk behavior, which may be more vulnerable to underreporting; this study used a more comprehensive composite index combining multiple behaviors, which was considered a more accurate measure than any single dichotomous indicator (Williams et al., 2001). All four psycho-socio-behavioral characteristics themselves remained significant and independent correlates of sexual risk behavior. Lonely feelings or social isolation, lax social control, and perceived sexual risk behaviors among social network members were all associated with more sexual risk behaviors.

Some limitations of the study are worth noting. First, the research setting is not representative of China's more developed coastal provinces. Second, some registration files used in classifying individuals into the four groups in the third stage of the sampling may not be complete, e.g. not every temporary migrant, drug user, HIV-positive person would have been registered, However, the grouping was used only to over sample the rare population elements (i.e. HIV-positive persons, drug users, and temporary migrants) and did not have to be complete in coverage. Any missing rare populations from their respective registrations would have been included in the non-migrant group/list; their HIV, drug use, and migrant status would have been reported in the survey questionnaire. Third, the data set is a little outdated. Given the rapid social and economic changes in China, what the data reveal more than 10 years ago may no longer be representative today. Fourth, data collection is based on self-reporting. Many factors may affect the accuracy of self-reporting, including memory lapse, social desirability, and knowledge about family members' and peers' sexual behaviors. And fifth, the study design is cross-sectional, which makes it impossible to pinpoint the exact causal relationship between migration and migrants’ mental and sexual health or any potential mediating mechanism.

With these study limitations in mind, the results suggest that rural–urban temporary migrants are at risk of mental and sexual health problems. Although rural–urban migrants are subject simultaneously to the influence of migration as a process that induces psycho-socio-behavioral changes and post-migration urban living, our results indicate that the influence of migration as a process is more important than urban living in understanding migrants’ mental health problems and sexual risk behavior. The lack of any significant difference by migrants' rural vs. urban origin may be further evidence that the process of migration plays a more important role than post-migration urban living or pre-migration residence. Migration-induced changes in psycho-socio-behavioral characteristics may very well mediate the relationship between migration and migrants’ mental health and sexual risk behavior. In addition, the selectivity of migration may also predispose migrants to or make them vulnerable to mental health problems and sexual risk behavior. Rural–urban migrants’ mental and sexual health problems deserve more immediate research attention. Both behavioral and intervention research are needed to better understand the migration-health links and to mitigate the detrimental impact of migration on migrants’ health. If results from this study are of any indication, health prevention programs need to identify the right target population for the right health problems. For example, this study finds males may be more vulnerable to sexual health problems while females more vulnerable to mental health problems. To be effective, health prevention programs also need to be better informed by knowledge about potential mechanisms, such as social isolation and lax social control, which may mediate the impact of migration on migrants’ health.

## References

[CIT0001] Anderson A., Qingsi Z., Hua X., Jianfeng B. (2003). China's floating population and the potential for HIV transmission: A social-behavioural perspective. *AIDS Care*.

[CIT0002] Bandura A. (1986). *Social foundations of thought and action: A social cognitive theory*.

[CIT0003] Berhan Y., Berhan A. (2013). Meta-analysis on risky sexual behavior of men: Consistent findings from different parts of the world. *AIDS Care*.

[CIT0004] Bhugra D. (2004). Migration and mental health. *Acta Psychiatrica Scandinavica*.

[CIT0005] Bilsborrow R., Hugo G., Oberai A., Zlotnik H. (1997). *International migration statistics: Guidelines for the improvement of data collection systems*.

[CIT0006] Chan K. W. (1996). Post-Mao China: A two-class urban society in the making. *International Journal of Urban and Regional Research*.

[CIT0007] Chan K. W., Liu T., Yang Y. (1999). Hukou and non-Hukou migrations in China: Comparisons and contrasts. *International Journal of Population Geography*.

[CIT0008] Chen J. (2011). Internal migration and health: Re-examining the healthy migrant phenomenon in China. *Social Science and Medicine*.

[CIT0009] Clark N. (1987). Social learning theory in current health education practice. *Advances in Health Education and Promotion*.

[CIT0010] Cohen D., Spear S., Scribner R., Kissinger P., Mason K., Wildgen J. (2000). “Broken windows” and the risk of gonorrhea. *American Journal of Public Health*.

[CIT0011] Cui X., Rockett I., Yang T., Cao R. (2012). Work stress, life stress, and smoking among rural-urban migrant workers in China. *BMC Public Health*.

[CIT0012] Emler N., Derlega V., Winstead B., Jones B. (2005). Moral character. *Personality: Contemporary theory and research*.

[CIT0013] Evans G. W. (2003). The built environment and mental health. *Journal of Urban Health: Bulletin of the New York Academy of Medicine*.

[CIT0014] Frye V., Latka M., Koblin B., Halkitis P., Putnam S., Galea S., Vlahov D. (2006). The urban environment and sexual risk behaviour among men who have sex with men. *Journal of Urban Health: Bulletin of the New York Academy of Medicine*.

[CIT0015] Galea S., Ahern J., Vlahov D. (2003). Contextual determinants of drug use risk behaviour: A theoretic framework. *Journal of Urban Health: Bulletin of the New York Academy of Medicine*.

[CIT0016] Galea S., Freudenberg N., Vlahov D. (2005). Cities and population health. *Social Science and Medicine*.

[CIT0017] Galea S., Uddin M., Koenen K. (2011). The urban environment and mental disorders: Epigenetic links. *Epigenetics*.

[CIT0018] Galea S., Vlahov D. (2005). Urban health: Evidence, challenges, and directions. *Annual Review of Public Health*.

[CIT0019] Gil V., Wang M., Anderson A., Lin G., Wu Z. (1996). Prostitutes, prostitution and STD/HIV transmission in Mainland China. *Social Science and Medicine*.

[CIT0020] He X., Wong D. F. K. (2013). A comparison of female migrant workers’ mental health in four cities in China. *International Journal of Social Psychiatry*.

[CIT0021] Hesketh T., Ye X. J., Li L., Wang H. M. (2008). Health status and access to health care of migrant workers in China. *Public Health Report*.

[CIT0022] Hyde S. (2000). Selling sex and sidestepping the state: Prostitutes, condoms, and HIV/AIDS prevention in southwest China. *East Asia: An International Quarterly*.

[CIT0023] Jochelson K., Mothibeli M., Leger J. (1991). Human immunodeficiency virus and migrant labor in South Africa. *International Journal of Health Services*.

[CIT0024] Knight J., Song L., Jia H. (1999). Chinese rural migrants in urban enterprises: Three perspectives. *Journal of Development Studies*.

[CIT0025] Latkin C., Forman V., Knowlton A., Sherman S. (2003). Norms, social networks, and HIV-related risk behaviors among urban disadvantaged drug users. *Social Science and Medicine*.

[CIT0026] Li L., Wang H., Ye X., Jiang M., Lou Q., Hesketh T. (2007). The mental health status of Chinese rural–urban migrant workers: Comparison with permanent urban and rural dwellers. *Social Psychiatry and Psychiatric Epidemiology*.

[CIT0027] Li X., Stanton B., Fang X., Lin D. (2006). Social stigma and mental health among rural-to-urban migrants in China: A conceptual framework and future research needs. *World Health and Population*.

[CIT0028] Liang Z., Ma Z. (2004). China's floating population: New evidence from the 2000 census. *Population and Development Review*.

[CIT0029] Lin D., Li X., Wang B., Hong Y., Fang X., Qin X., Stanton B. (2011). Discrimination, perceived social inequity, and mental health among rural-to-urban migrants in China. *Community Mental Health Journal*.

[CIT0030] Lu Y. (2008). Test of the “healthy migrant hypothesis”: A longitudinal analysis of health selectivity of internal migration in Indonesia. *Social Science and Medicine*.

[CIT0031] Lu Y. (2010). Mental health and risk behaviours of rural-urban migrants: Longitudinal evidence from Indonesia. *Population Studies*.

[CIT0032] Ma L., Xiang B. (1998). Native place, migration and the emergence of peasant enclaves in Beijing. *The China Quarterly*.

[CIT0033] Macintyre S., Maciver S., Sooman A. (1993). Area, class and health: Should we be focusing on places or people?. *Journal of Social Policy*.

[CIT0034] Mair C., Diez A. V. D., Galea S. (2008). Are neighbourhood characteristics associated with depressive symptoms? A review of evidence. *Journal of Epidemiology and Community Health*.

[CIT0035] Marsella A. (1998). Urbanization, mental health, and social deviancy: A review of issues and research. *American Psychologist*.

[CIT0036] Moore M., Gould P., Keary B. (2003). Global urbanization and impact on health. *International Journal of Hygiene and Environmental Health*.

[CIT0037] National Bureau of Statistics of China (2012). *China statistical yearbook 2012*.

[CIT0038] Paykel E., Abbott R., Jenkins R., Brugha T., Meltzer H. (2000). Urban-rural mental health differences in Great Britain: Findings from the national morbidity survey. *Psychological Medicine*.

[CIT0039] Peen J., Schoevers R. A., Beekman A. T., Dekker J. (2010). The current status of urban-rural differences in psychiatric disorders. *Acta Psychiatrica Scandinavica*.

[CIT0040] Radloff L. (1977). The CES-D Scale: A self-report depression scale for research in the general population. *Applied Psychological Measurement*.

[CIT0041] Rechel B., Mladovsky P., Ingleby D., Mackenbach J. P., McKee M. (2013). Migration and health in an increasingly diverse Europe. *Lancet*.

[CIT0042] Richard A., Bell D., Montoya I. (2000). Normative influence on condom use in the personal networks of female cocaine smokers. *AIDS Education and Prevention*.

[CIT0043] Rosen G. (1993). *A history of public health*.

[CIT0044] Solinger D. (1999). *Contesting citizenship in urban China: Peasant migrants, the state, and the logic of the market*.

[CIT0045] Sudhinaraset M., Mmari K., Go V., Blum R. (2012). Sexual attitudes, behaviours and acculturation among young migrants in Shanghai. *Culture, Health and Sexuality*.

[CIT0046] Wang B., Li X., Stanton B., Fang X. (2010). The influence of social stigma and discriminatory experience on psychological distress and quality of life among rural-to-urban migrants in China. *Social Science and Medicine*.

[CIT0047] Wang F. (2004). Reformed migration control and new targeted people: China's hukou system in the 2000s. *The China Quarterly*.

[CIT0048] Wang F., Zuo X., Ruan D. (2002). Rural migrants in Shanghai: Living under the shadow of socialism. *International Migration Review*.

[CIT0049] Wang W., Wei C., Buchholz M. E., Martin M. C., Smith B. D., Huang Z. J., Wong F. Y. (2010). Prevalence and risks for sexually transmitted infections among a national sample of migrants versus non-migrants in China. *International Journal of STD and AIDS*.

[CIT0050] Weiss R., McMichael A. (2004). Social and environmental risk factors in the emergence of infectious diseases. *Nature Medicine Supplement*.

[CIT0051] Wen M., Wang G. (2009). Demographic, psychological, and social environmental factors of loneliness and satisfaction among rural-to-urban migrants in Shanghai, China. *International Journal of Comparative Sociology*.

[CIT0052] Williams M., McCoy H. V., Bowen A., Saunders L., Freeman R., Chen D. (2001). An evaluation of a brief HIV risk reduction intervention using empirically derived drug use and sexual risk indices. *AIDS and Behavior*.

[CIT0053] Wong D. F. K., Chang Y. L., He X. S. (2007). Rural migrant workers in urban China: Living a marginalized life. *International Journal of Social Welfare*.

[CIT0054] Wong D. F. K., He X., Leung G., Lau Y., Chang Y. (2008). Mental health of migrant workers in China: Prevalence and correlates. *Social Psychiatry and Psychiatric Epidemiology*.

[CIT0055] Yang T., Xu X., Li M., Rockett I. R. H., Zhu W., Ellison-Barnes A. (2012). Mental health status and related characteristics of Chinese male rural-urban migrant workers. *Community Mental Health Journal*.

[CIT0056] Yang X. (1993). Household registration, economic reform, and migration. *International Migration Review*.

[CIT0057] Yang X. (2005). Does where we live matter? Community characteristics and HIV/STD prevalence in southwestern China. *International Journal of STD and AIDS*.

[CIT0058] Yang X. (2006). Temporary migration and HIV risk behaviors in China. *Environment and Planning A*.

[CIT0059] Yang X., Derlega V., Luo H. (2007). Migration, behavior change, and HIV/STD risks in China. *AIDS Care*.

[CIT0060] Yang X., Luo H. (2009). Migration, urbanization, and drug use and casual sex in China: A multilevel analysis. *Environment and Planning A*.

[CIT0061] Yang X., Xia G. (2006). Gender, migration, risky sex, and HIV infection in China. *Studies in Family Planning*.

[CIT0062] Yang X., Xia X. (2008). Temporary migration and STD/HIV risk sexual behavior: A population-based analysis of gender differences in China. *Social Problems*.

[CIT0063] Zhang L. (2001). *Strangers in the city: Reconfigurations of space, power, and social networks within China's floating population*.

[CIT0064] Zhang L., Chow E., Jahn H. J., Kraemer A., Wilson D. P. (2013). High HIV prevalence and risk of infection among rural-to-urban migrants in various migration stages in China: A systematic review and meta-analysis. *Sexually Transmitted Diseases*.

[CIT0065] Zheng W., Zhou X., Zhou C., Liu W., Li L., Hesketh T. (2011). Detraditionalisation and attitudes to sex outside marriage in China. *Culture, Health & Sexuality*.

